# Two Different Upper Tract Urological Malignancies on Either Side

**DOI:** 10.1155/2021/9981381

**Published:** 2021-09-21

**Authors:** W. S. L. De Silva, S. R. De Almeida, G. D. B. J. Karunarathne, A. A. S. Samarathunga, K. M. C. S. Gannoruwa, J. A. S. B. Jayasundara

**Affiliations:** ^1^Postgraduate Institute of Medicine, University of Colombo, Sri Lanka; ^2^District General Hospital, Nuwara Eliya, Sri Lanka

## Abstract

**Introduction:**

The genitourinary system is a recognized site for multiple primary malignant neoplasms even without syndromic anomalies. However, to the best of our knowledge, a case of upper tract urothelial carcinoma (UTUC) with contralateral renal cell carcinoma (RCC) is not reported in surgical literature so far. *Case Presentation*. A 52-year-old Sri Lankan male patient was found to have a right lower ureteric tumour and a left renal mass together upon investigating for painless visible hematuria. The right ureteric tumour measured 32 × 22 mm resulting in moderate hydronephrosis and cortical thinning of the right kidney, and the left renal mass measured 43 × 38 mm involving the lower pole. The biopsy of the right ureteric lesion revealed a high-grade transitional cell carcinoma with focal nested pattern and that of the left renal mass revealed a clear cell carcinoma. Right nephroureterectomy followed by a left partial nephrectomy was performed in six weeks' interval. The histology of both the resected specimens confirmed the biopsy findings. *Discussion*. A high-risk upper tract urothelial carcinoma such as the right ureteric tumour of this patient required a nephroureterectomy which makes the management of the contralateral renal cell carcinoma more complex. An adequate functional renal remnant was ensured after offering oncologically sound surgical treatment for both the malignancies of this patient.

**Conclusion:**

A UTUC when associated with a contralateral RCC poses challenges in patient management. The preservation of renal excretory function has to be considered as an important determinant in addition to oncologically sound surgical resection when managing complex cases of genitourinary malignancies involving both sides of the upper urinary tract.

## 1. Introduction

The phenomenon of multiple primary malignant neoplasms in nonsyndromic individuals was first described by Billroth way back in 1889 [1]. Moertel et al. detailed the criteria for the diagnosis of synchronous multiple primary malignancies (SMPM) later in 1961 [2]. The male genitourinary system (GUS) is considered as one of the commonest systems to harbor SMPM. Hence, the term synchronous primary urological cancer (SPUC) was coined by Qin et al. [3]. The knowledge about the behavior of each malignancy and the overall outcome of patients having SPUC is evolving. The overall prognosis of patients with SPUC is observed to be unfavorable to that of patients having a solitary urological malignancy, owing to the higher grade and the stage of tumours at presentation [3]. The management of patients with SPUC is challenging, especially when it involves the upper GUS because of the requirement to preserve an adequate functional renal remnant despite the advanced stage of the disease at presentation. We report a case of a high-risk upper tract urothelial carcinoma (UTUC) with a contralateral renal cell carcinoma (RCC) which required a patient-tailored management approach.

## 2. Case Presentation

A 52-year-old male was referred by the general practitioner for the management of an ultrasonographically detected left renal mass upon investigation for a single episode of frank hematuria. There was no history of loin pain, back pain, or lower urinary tract symptoms. He was a smoker (40 pack-years) but had no comorbidities. He had neither a personal history of malignancies elsewhere nor a history of cancer syndrome such as Lynch syndrome in the family. He had two subsequent bouts of frank hematuria in the preceding week and was admitted. No loin mass was palpable per-abdominally. The prostate gland was soft and not enlarged clinically, and the rest of the physical examination was unremarkable. The hemoglobin level on admission was 7.2 g/dl, and he was transfused accordingly. Other basic haematological and biochemical investigations were within normal limits. His baseline serum creatinine (S.Cr) was 0.9 mg/dl, and his serum prostate specific antigen level was 0.2 ng/ml.

**Figure 1:**
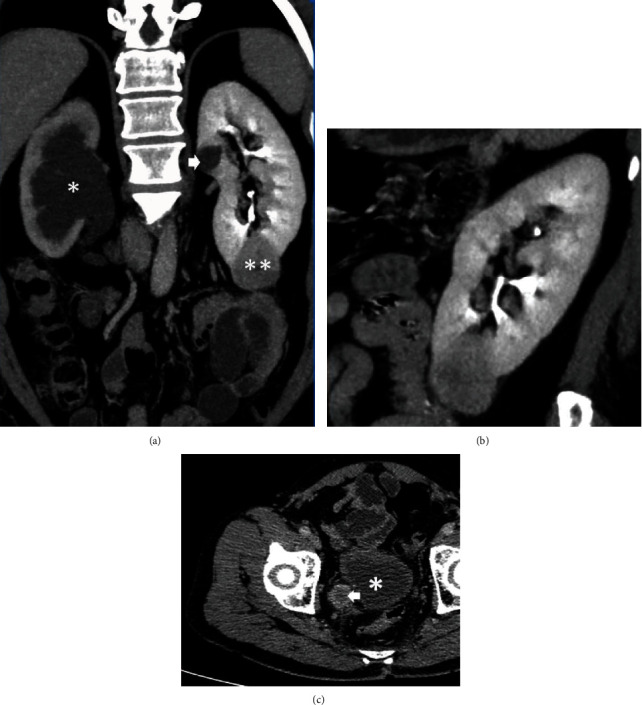
CT images of the patient. (a) A coronal section illustrating the hydronephrotic right kidney (∗) due to the obstruction caused by the lower ureteric tumour and the left kidney with a contrast enhancing mass in the lower pole (∗∗) and a simple cyst in the upper pole (arrow). (b) A coronal section through the left renal mass. (c) An axial section showing the right ureteric tumour (arrow) in relation to the posterior wall of the bladder (∗).

**Figure 2:**
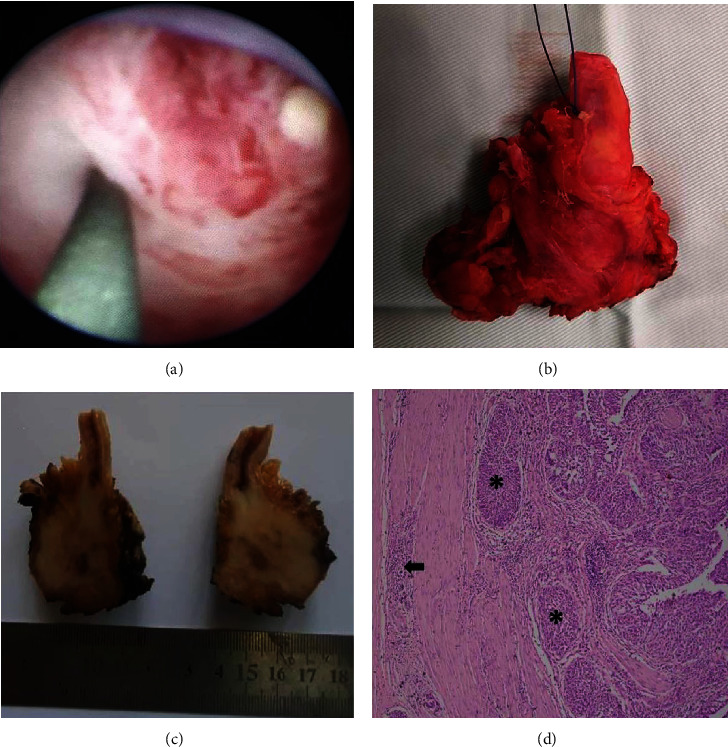
Right ureteric tumour. (a) Ureteroscopic view of the tumour completely obstructing the lumen of the ureter. (b) Macroscopic view of the separately resected tumour with a bladder cuff. (c) Cut surface appearance of the tumour. (d) ×10 view illustrating the nests of malignant (∗) cells invading the muscularis propria of the ureter (arrow).

The computerized tomographic urogram revealed an enhancing mass lesion measuring 32 × 22 mm in the right lower ureter, resulting in moderate hydronephrosis of the right kidney with a cortical thickness of 12 mm. In the left kidney, there was an enhancing, exophytic mass, measuring 43 × 38 mm involving the lower pole (Nephrometry/RENAL score 5a) (Figure 1). There was no abdominal lymphadenopathy or evidence of metastasis. The differential function of the two kidneys was right—35% and left—65%. Flexible cystoscopy revealed no synchronous bladder lesions. The ureteroscopic biopsy of the lesion in the right lower ureter revealed a high-grade urothelial carcinoma with focal nested pattern, and the image-guided biopsy of the left renal mass confirmed a clear cell renal cell carcinoma (ccRCC). Following multidisciplinary discussion, right nephroureterectomy with removal of a bladder cuff was performed initially. A single bladder instillation of mitomycin C was also offered the same day. Histology of the specimen confirmed a high-grade UTUC with extensive lymphovascular and perineural invasion. All resection margins and three local lymph nodes were negative for malignancy. However, there was evidence of invasion of periureteric fat (pT_3_N_0_M_0_) (Figure 2).

**Figure 3:**
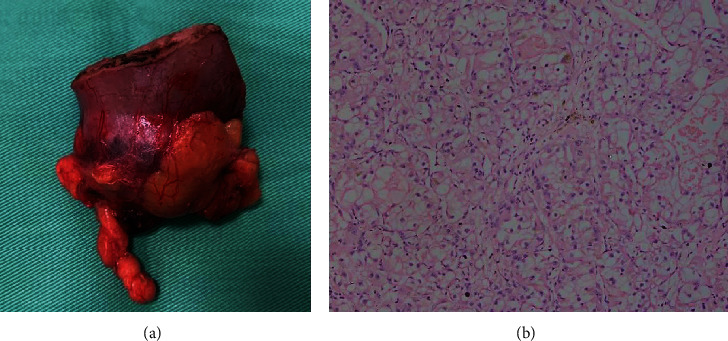
Left renal cell carcinoma. (a) Macroscopic appearance of the resected tumour. (b) Histology showing typical clear cell carcinoma.

Left open partial nephrectomy was performed six weeks later. Histology of which revealed a low-grade (ISUP grade 2) ccRCC without sinus fat or capsular involvement, and the resection margins were negative (pT_1b_N_x_M_0_) (Figure 3). Following the partial nephrectomy, the patient went into a status of acute kidney injury (AKI) where S.Cr raised to 4.5 mg/dl. Despite the biochemical derangement, he maintained a good urine output throughout and recovered from AKI in two weeks without requiring any renal replacement therapy and S.Cr came down to 1.1 mg/dl. The patient was given six cycles of cisplatin-based combination chemotherapy without detectable toxicity. No recurrence of either malignancy was detected at one-year follow-up. He is scheduled for annual CT imaging and three monthly clinic follow-up.

## 3. Discussion

Many reported cases of SPUC involve the bladder and the prostate gland [4]. 23% to 54% of patients who undergo radical cystoprostatectomy for bladder cancer were found to have synchronous prostate cancer [5]. Several cases of bladder cancers associated with RCC are reported [6, 7]. Two sporadic urological cancers involving the upper GUS are very rare. A case of a RCC and an ipsilateral TCC in the renal pelvis was reported by Lu et al. [8]. In this case, both malignancies were found in the same kidney which makes it less complex to management. To the best of our knowledge, this is the first case to be reported with a UTUC with a contralateral RCC.

Clinical guidelines recommend nephroureterectomy for a high-risk UTUC as the right-sided malignancy the index patient had [9]. However, the management of the index case was complicated due to the presence of the contralateral RCC, which again requires partial nephrectomy with a clear margin. A surgeon should be satisfied with the renal remnant to maintain the excretory function when planning this type of cases. Transient impairment of the renal function should be expected in a case like this because of the surgical trauma to the remnant kidney. However, there needs to be a contingency plan if an unexpected intra/postoperative complication makes the patient anephric. Preoperative planning, counseling, and consenting for such potential unfortunate events are mandatory in the management of rare cases of bilateral SPUC like the index case. Also, the involvement of specialties such as oncology, pathology, nephrology, and anesthesia is critical in tailor mating the treatment for puzzling cases like the index case.

Long-term follow-up of patients with SPUC is also pivotal because of high risk of recurrence due to higher stage and grade of individual malignancies. Recurrence from both malignancies was reported in the above discussed case by Lu et al. [8], emphasizing the requirement of special follow-up protocols for the long-term management of patients with SPUCs.

## 4. Conclusion

A UTUC when associated with a contralateral RCC poses several challenges in patient management. Staged ipsilateral nephroureterectomy and contralateral partial nephrectomy can be performed safely while preserving renal excretory functions.

## Data Availability

All data pertaining to this article are available with the authors.
